# Five Essays

**Published:** 1859-07

**Authors:** 


ART. VI.—*Five Essays.* By J. K. MITCHELL, M.D, etc. etc. Edited by S. WEIR MITCHELL, M.D., etc. 12mo., pp. 371. J. B. Lippincott & Co. 1859.

THIS tribute of filial piety to the memory of a distinguished father contains much that is worthy of note and preservation.

The first of the selected essays, "On the Cryptoganous Origin of Malarious and Epidemic Fevers," has been for some years before the public, attracting large consideration and comment. It is not too high praise to say of it that it is the most ingenious and able attempt that has been made at the elucidation of the difficult topic, whose obscurity has indeed bid defiance to the accumulated efforts of professional intelligence.

We think the riddle still unsolved. There are capricious incongruities in the history of this class of endemics and local epidemics which do not admit of any comfortable or satisfactory explanation in the present condition of science. We do not hesitate to affirm, however, that there is no monograph on malaria, no treatise on the etiology of that large class of maladies which we ascribe to a specific poison, referred to most vaguely under that title, which the student can peruse with more instruction, and the pathologist with more curious and exciting interest. We are very glad to see it reissued in a permanent and becoming form.

The second essay, the longest in the book, upon "Animal Magnetism, or Vital Induction," is now published for the first time. We need not assure our readers that it will abundantly reward the most careful attention devoted to its perusal. It is a great mistake to imagine that the study of such subjects can be evaded by the physician. "Nihil humanum mihi alienum puto," must be the motto of every one of us. Whatever concerns, whatever interests our clients, must be brought under our contemplation. From the earliest times, some latent power of the nature here investigated has been believed to exist, and believed not only by the vulgar and ignorant, but by the shrewdest men of the world, the most intelligent observers of facts, the most profound cultivators of science. Like many other articles of time-honored and universal faith, it has been found no easy matter to express it in any acceptable formula, to choose the proper words for a creed or dogma on which men could agree. But as to the fact that there is *something to believe,* although we cannot precisely define it, there can be no question. The January number of the *Westminster Review* contains an article on anæsthetics, evidently from an able and practical pen, in which this matter is set in a strong light, and spoken of as an undoubted verity.

Mesmerism is treated of only as an anæsthetic agent; but its potency, effectiveness, applicability, and its subjection to certain laws, are regarded as absolutely established. And indeed it is idle to deny, that when every allowance is made for delusion and collusion, error and knavery, there is still "a residuum," as some one has called it, which requires explanation, and has not yet been explained. "Imagination and imitation," says Prof. Mitchell, "cannot account for the uniformity of the mesmeric state in persons of all ages and conditions, who are totally ignorant not only of the symptoms to be produced, but of the design of the mesmerizer; nor can we, by any rational view of their causes, ascribe to anything *but a physical influence*, the effect of passes," etc. This physical influence he denominates "vital induction;" "an unexplained power, analogous to the equally inexplicable induction of the mechanicians, and the *presence* of the chemist."

It would indeed be a blind and deaf skepticism that would ignore at the present day the facts vouched for by so many individuals as matters of personal experience or observation; the statements of Agassiz, Esdaile, Dugas; the experiments of Mitchell, Mayo, and Elliottson; the recorded opinions of La Place, Cabanis, De Jussieu, and Cuvier.

There was no little courage required for the investigation entered into and so skillfully conducted by Prof. Mitchell. Our profession is exquisitely sensitive—and long may it continue so—as to contact with the numerous forms of charlatanism, even for the purposes of detection and exposure; and there never was, perhaps, any topic of human attention so thoroughly imbued with the very spirit of imposture as this. Its very name is painfully and palpably characteristic; and in its progress it seemed to put on darker and darker colors at every step. But Prof. Mitchell feared nothing; and his acuteness was equal to his courage. The inquiry could not possibly have fallen into more competent hands; and the result, as here recorded, justified fully all the expectations of his most sanguine friends.

Of the two purely scientific papers, "On the Penetrativeness of Fluids," and "On the Penetration of Gases," it is only proper to remark, that they were well-timed, somewhat in advance of the philosophy of the day, which they aided to lead, and to the progress of which they largely and impressively contributed. The reputation accruing from them was great, and is destined to last.

The concluding paper, "On a New Practice in Acute and Chronic Rheumatism," was happily chosen by the editor as a fair specimen of the numerous and valuable practical suggestions for which the medical profession is indebted to Professor Mitchell.

The volume before us affords abundant proofs of his possession of a rare combination of intellectual qualities. Genius of a high order, a quick perceptive capacity, a most prompt apprehension and comparison, were mingled in him with profound and cautious reflection, and a plain, simple, and practical adaptation of means to ends; all which go to constitute the highest sagacity of the physician, and the most extended usefulness of the man of administrative talent.

The work is gotten up in the admirable style with which the enterprising and liberal publishers, J. B. Lippincott & Co., have made us familiar, and for which they are justly celebrated. It must hereafter form a portion of the library, not only of each member of the widely-distributed body of alumni of the Jefferson Medical College, to whom it is gracefully and feelingly dedicated, but to every American physician.

